# Understanding the chemical and mineralogical composition of commercial henna and jagua tattoos and dyes—a multi-analytical approach

**DOI:** 10.1007/s00216-022-04194-1

**Published:** 2022-07-13

**Authors:** Laura Rubio, Mafalda Costa, Pedro Barrulas, Marta Lores, Carmen Garcia-Jares, Cristina Barrocas-Dias

**Affiliations:** 1 CRETUS, Department of Analytical Chemistry, Nutrition and Food Science, Universidade de Santiago de Compostela, 15782 Santiago de Compostela, Spain; 2 Laboratory of Research and Development of Analytical Solutions (LIDSA), Department of Analytical Chemistry, Nutrition and Food Science, Universidade de Santiago de Compostela, 15782 Santiago de Compostela, Spain; 3 HERCULES Laboratory, University of Évora, Palácio do Vimioso, Largo Marquês de Marialva 8, 7000-809 Évora, Portugal; 4 Chemistry and Biochemistry Department, School of Sciences and Technology, Rua Romão Ramalho, 59, 7000-671 Évora, Portugal

**Keywords:** Henna, Jagua, Natural pigments, h-XRF, XRD, VP-SEM–EDS

## Abstract

Temporary tattoos and dyes constitute a great analytical challenge in relation to the regulatory control of their ingredients. Most of these commercial products are not labeled according to their content and their chemical nature is highly diverse. Therefore, it is necessary to analyze these complex samples to evaluate the potential presence of metallic impurities, to ensure the safety of cosmetic products contributing to health protection. This study proposes a multi-analytical methodology, which includes handheld X-ray fluorescence (h-XRF) and X‐ray diffraction (XRD), complemented by variable pressure scanning electron microscopy coupled with energy-dispersive X‐ray spectrometry (VP-SEM–EDS) to fully characterize 34 commercial samples of jagua and henna tattoos and dyes. The approach allowed the identification of the main constituents providing complementary compositional data and differences between sample types were established. In addition, information on the degree of natural pigments homogeneity was also obtained. The results’ discussion considering the current European cosmetics regulation may be useful to support the drafting of safety requirements and specific regulation.

**Supplementary Information:**

The online version contains supplementary material available at 10.1007/s00216-022-04194-1.

## Introduction

Henna has been widely used since ancient times as a traditional body decoration and natural remedy in many parts of the world. Henna is also applied as temporary hair dye or paint-on tattoos on the hands and feet for cosmetic purposes, thus providing an alternative to permanent products. In recent years, there has been a renewed trend for plant-based cosmetics and personal care products [1], and in addition, other alternative dyes have appeared, such as jagua temporary tattoos, which are often sold on the Internet and use natural pigments in a similar way to henna tattoos.

Henna is a brown or green finely ground powder obtained from dried leaves of the plant *Lawsonia inermis* (*Lythraceae* family), which is grown in dry tropical and sub-tropical areas, such as North Africa, India, Sri Lanka, and the Middle East. The traditional plant–derived henna contains the active ingredient called lawsone (2-hydroxy-1,4-naphthoquinone), which is an orange-red pigment responsible for the typical coloration [2]. Lawsone interacts directly with the amine or sulfur functional groups of keratin due to its strong affinity. Henna is usually mixed with water or essential oils to make a paste that is applied as a hair dye or to decorate the hands and feet.

Jagua, which was already used by the indigenous tribes to stain the skin, is obtained from an Amazonian tropical fruit known as *Genipa americana* L. (*Rubiaceae* family). The dye comes from the sap of an unripe fruit, and it turns dark blue or blackish when it is applied to the body. Jagua contains main ingredients geniposide and its bioactive derivative, genipin, which have been mentioned in some studies suggesting that they may become potential new allergens in temporary tattoos [3, 4]. When genipin, which is a colorless substance, comes into contact with the skin, it immediately reacts with the present proteins to give the pigment color.

Natural and anthropogenic factors such as cultivation in contaminated areas [5, 6] as well as further processing of the plants can greatly affect the final chemical composition of these natural pigments. In addition, the degradation of the compounds they contain [7] can also occur due to different factors. More recently, henna and jagua preparations have been fortified with various herbs, or natural or synthetic chemical materials to modify or enhance color, and improve dye penetration and fixation, among other reasons. It is suspected that the added material includes several mineral products which are very rich in heavy metals [8] and the mixtures performed could further increase their potential original concentrations. Although the mechanism of skin interaction is related to the active ingredients that provide the typical colorations, it is true that other compounds, such as certain metals, may contribute or have a synergistic action in this coloration.

Temporary tattoos are products with a diverse chemical composition and unclear legislation [9, 10]. Both types abovementioned can be included in the group of new-format cosmetics or considered *borderline* products in the European regulations [11]. Under the guidelines of the Manual of Borderline Products, they should meet both the Cosmetics European Regulation EC No 1223/2009 [12] and the Toys Directive [13]. EC legislation is the main regulatory framework for marketed cosmetic products and it lists ingredients that are allowed, restricted under certain conditions, or even banned throughout the different annexes. However, the reality is far from fulfilling these requirements. The regulations on natural pigment-based temporary tattoos have not yet been published and most of these commercial products are not labeled. The safety of henna and its main active ingredient, lawsone, has been evaluated in hair dye products [14], concluding safe use as a hair dye in specific formulations. Nevertheless, no such evaluation has yet been performed for other similar products containing henna extracts, such as temporary tattoos. Jagua-based tattoos have not even been considered from a regulatory point of view, although their allergenic potential has already been evidenced. The safety of these products is highly questionable and their potential to cause skin reactions should be considered.

If we focus on the selected samples, there is only limited research in the literature that applied spectrometric techniques. They have been applied to henna samples in separate and incomplete analyses. However, there is no published work on jagua products, so this is the first time this methodology has been proposed.

In this study, selected commercially available plant-based products used as temporary tattoos and hair dyes were analyzed using a multi-analytical methodology, in order to gain new information regarding their chemical and mineralogical composition. The results obtained using handheld X-ray fluorescence (h-XRF), X-ray diffraction (XRD), and variable pressure scanning electron microscopy coupled to energy-dispersive X-ray spectrometry (VP-SEM–EDS) were discussed taking into consideration their use in the cosmetics and pharmaceutical industries and the current European safety regulations. The final objective is to obtain useful knowledge and information in order to help steer towards both the establishment of safety standards in the manufacturing process and the publication of a correct and specific regulation for temporary cosmetics.

## Material and methods

### Samples

A total of 34 commercial samples were considered, comprising two different types: temporary jagua tattoos (6 samples, abbreviated as JT) and henna-based products (27 in total). These henna samples can be divided into different classes according to their purpose: temporary tattoos (17, HT), hair dyes (7, HD), and henna samples for both claims (3, HTD). All samples have been numbered correlatively. An additional plant-based temporary tattoo sample (HPT) was also included and analyzed in a general way with the jagua group although its composition and origin were unknown. Supplementary Table S1 shows images of the 34 samples purchased.

The samples can also be divided into two groups according to their physical state: paste (23) and solid (11). Regarding the color of the samples, some have typical colors, but others are more striking. The color and sample type are detailed in Table 1.

Among the selected henna products, two samples were collected from a local source in Morocco (HT-13 and HT-14) and the rest were acquired through a well-known site available to anyone on the Internet from different sellers and at a very accessible price. Concerning jagua samples, all those that were available at the moment were also purchased online. All samples were kept in their original containers protected from light and stored at room temperature until analysis.

**Table 1:** Type and color of the analyzed samples

Sample name	Color
Sample type	
Solid	
HD-1	Greenish
HD-2	
HD-3	
HD-4	
HD-5	
HD-6	
HD-7	
HTD-1	
HTD-2	
HTD-3	
JT-4	Black
Paste	
HT-1	Black
HT-2	Red
HT-3	White
HT-4	Orange
HT-5	Pink
HT-6	Reddish
HT-7	Green
HT-8	Blue
HT-9	Violet
HT-10	Black
HT-11	Brown
HT-12	Red
HT-13	Black
HT-14	Red
HT-15	Black
HT-16	Brown
HT-17	Blue
JT-1	Black
JT-2	Black
JT-3	Black
JT-5	Dark blue – Black
JT-6	Dark blue – Black
HPT	Black

*HT,* Henna Tattoo; *HD,* Henna Dye; * HTD,* Henna Tattoo-Dye; *JT,* Jagua Tattoo; *HPT,* Herbaceous Plant Tattoo

### Sample preparation

No sample preparation was performed for the solid samples. On the other hand, a couple of drops of the samples belonging to the paste group were air-dried at least 48 h before the measurements. For the SEM–EDS analysis, paste samples were dried directly on a thin conductive film of carbon over the glass slides that were subsequently introduced into the instrument.

### Instrumentation

#### Handheld X-ray fluorescence (h-XRF)

A handheld XRF Bruker™ Tracer III SD® with a silicon-drift (SDD) detector (XFlash®) and a Rh target delivering a polychromatic X-ray beam of 3 × 3 mm was used for XRF analysis. Spectra were recorded using a voltage of 40 kV and a current intensity of 11 μA was applied during a 180-s real-time count. All spectra were recorded using the S1PXRF software (Bruker™) and processed using the Artax software (Bruker™) in order to obtain semiquantitative data. The generated net areas of the fluorescence lines were averaged and normalized to the total count of the spectra [15].

#### X‐ray diffraction (XRD)

X-ray powder diffraction (XRPD) and micro-X-ray diffraction (μ-XRD) were used to identify the crystalline phases present in the solid and paste samples, respectively. Analysis was conducted using a Bruker™ D8 Discover® instrument equipped with a Cu Kα source and a LYNXEYE linear detector. All measurements were performed over a range from 3° to 75° 2θ, using a 0.05° increment. Measuring time from XRPD was generally 2 s/step but it was increased to 18 s per step to increase peak intensity in samples HD-2, HD-7, and HD-6. A zero-background holder, a 0.3 mm collimator, and a measuring time of 2 s/step were used to analyze the previously air-dried paste samples by μ-XRD. In this case, the measuring time was increased to 4 (HT-11, HT-14, and HPT), 6 (HT-9, HT-17, JT-2, and JT-5), or 10 (HD-3) seconds per step to increase peak resolution and intensity. The identification of all crystalline phases was performed with the DIFFRAC.SUITE EVA® software using the Powder Diffraction File (PDF‐2) X‐ray patterns database of the International Centre for Diffraction Data.

#### Variable pressure scanning electron microscope coupled with energy-dispersive X-ray Spectrometry (VP-SEM–EDS)

VP-SEM–EDS analysis was carried out using a Hitachi™ S3700N SEM coupled to a Bruker™ XFlash 5010 SDD EDS Detector® with an energy resolution of 129 eV in the MnKα line. The two types of samples were placed on a thin conductive carbon film and analyzed using a low vacuum (40 Pa) and with an accelerating voltage of 20 kV. The variable pressure approach allows for morphological characterization and performs point analysis and elemental mapping without the need for conductive coating materials. The chemical analysis obtained was used to complement the results of h-XRF and XRD. The compositional data was acquired using the Esprit1.9 software and a standardless quantification. The SEM images were acquired in the backscattering mode [16].

## Results and discussion

Due to the number of samples and analytical techniques used in this study, this section has been sub-divided according to the methodology used.

### Screening by h-XRF

Handheld XRF was used to obtain semiquantitative information on the bulk chemical composition of all the samples studied. This technique has proven to be particularly useful to establish compositional groups and to uncover affinities between chemical elements [15].

**Fig. 1:**
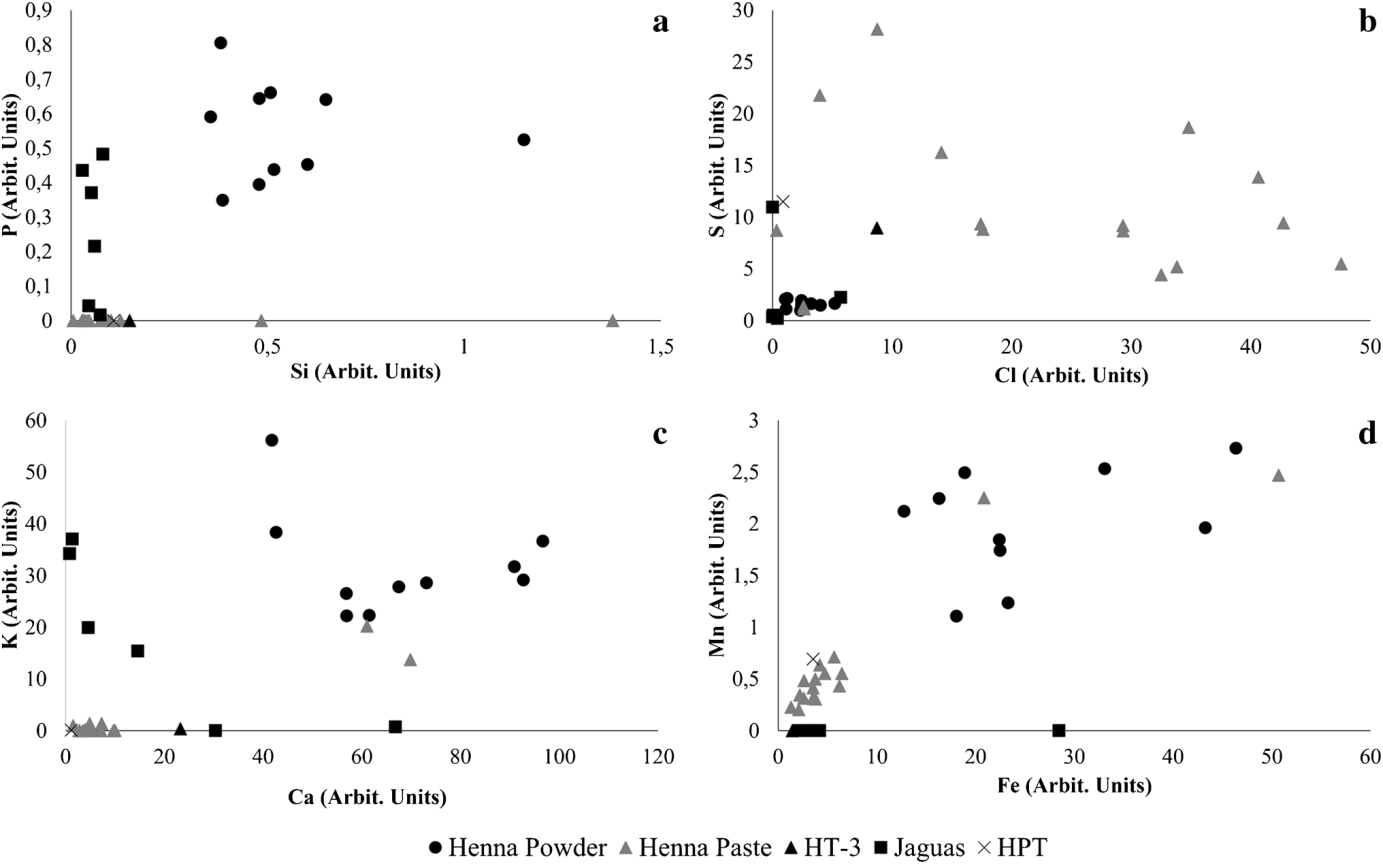
Elemental bi-plots evidencing compositional differences between the samples analyzed by h-XRF. **a** P-Si bi-plot evidencing the high Si contents in the henna powder samples and henna pastes HT-11 and HT-16, and the presence of P in all henna products; **b** S-Cl bi-plot showing the highest values of these elements can be found in henna paste samples; **c** K-Ca bi-plot indicating that these two elements are present in significant amounts in the henna powder samples and in henna pastes HT-11 and HT-16 and jagua samples JT-5 and JT-6; **d** Mn-Fe bi-plot evidencing variable iron contents in all samples, while manganese was only detected in henna samples and in sample HPT

Aluminum, Si, S, Cl, K, Ca, Ti, and Fe were detected in all samples, but, nevertheless, there are clear compositional differences between the different types of samples. In general, henna-powered samples have higher silicon contents than the henna pastes, the jaguas, or the plant-based temporary tattoo sample HPT. However, the henna pastes HT-11 and HT-16 also have significant Si values, similar or, in the case of sample HT-11, higher than the henna powders (Fig. 1a). Phosphorous was only detected in the henna powdered samples and in the jaguas, while sulfur and chlorine contents are generally higher in the henna pastes (Fig. 1a and b). In general, henna powdered samples and samples HT-11 and HT-16 have the highest K and Ca values; yet, samples HT-3, JT-5, and JT-6 also have relevant Ca contents, while the remaining jagua samples contain important amounts of K (Fig. 1c). Aluminum values were found to be similar in all samples (data not shown). On the other hand, iron contents, while variable, were higher in the henna-powered samples and in samples HT-11, HT-16, and JT-4 (Fig. 1d). Manganese was only detected in the hennas (with the exception of HT-3) and in sample HPT (Fig. 1d).

The similarity between the chemical composition of the henna powders and samples HT-11 and HT-16 should be noted and might be related to the fact that the active components of henna were only detected in these two henna paste samples in our previous works that explored the organic content and the presence of these bioactive ingredients in the samples [17, 18].

**Fig. 2:**
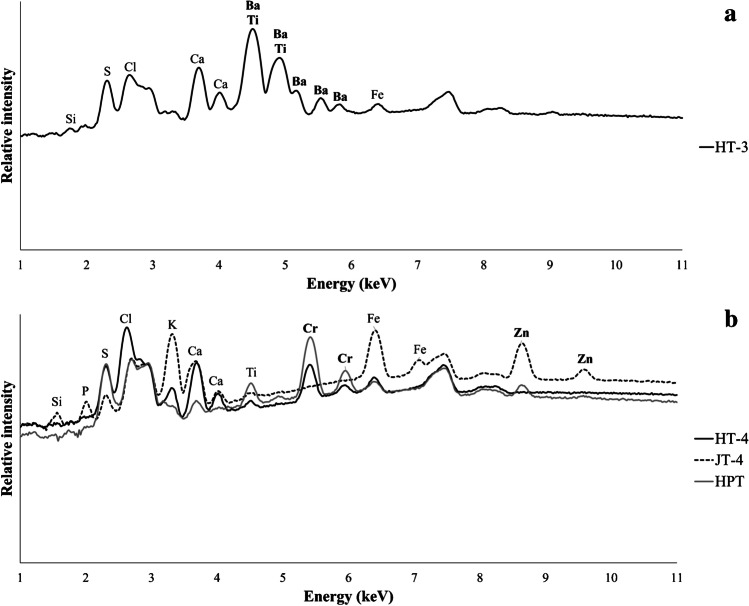
Handheld XRF spectra of selected samples: HT-3 (**a**), HT-4, JT-4, and HPT (**b**), evidencing the significant Ba and Ti contents detected in henna paste HT-3, the substantial Cr values found in samples HT-4 and HPT, and the high Zn content present in samples JT-4 and HPT

Significant Ti contents were detected in sample HT-3, along with Ba, which was only found in this sample (Fig. 2a). Given its color, and the high Ti and Ba values, the white colorants titanium dioxide (rutile) or barium sulfate (barite) widely used in cosmetic products [19, 20] may have been used to produce this henna paste.

Substantial Cr values were found in samples HT-4 and HPT, while high Zn content was uncovered in samples JT-4 and HPT (Fig. 2b). The high chromium values of samples HT-4 might explain its orange color as although the well-known inorganic Cr-based pigments have green hues, the monoazo pigments, consisting of a Cr complex, can result in red (Color Index number, CI, 18736) or yellow (CI 18690) colorants. High iron and manganese contents may also explain the greenish, brown, and black hues of the henna powders and samples HT-11, HT-16, and JT-4. In fact, natural earth pigments containing iron oxides and/or hydroxides or iron-rich clays can have a wide range of shades from deep purple and brown to red, yellow, and olive green [21], while manganese oxides are generally linked to brown colors [22].

Previous studies of henna products using XRF enabled the detection of elements such as Cd, Zn, Cu, Mn, Ni, and Co [23]. However, the authors could not conclusively attribute sample color to the presence of specific elements [23]. This is also true in the current study, with the exception of sample HT-3.

### Characterization by XRD

The results of the XRD analysis of the natural tattoo and dye samples are summarized in Table 2.

**Table 2:** Identification of the crystalline phases identified by XRD in the different types of samples: henna pastes, henna powders, and all jaguas

	*Henna pastes*																	*Henna powders*										*Jaguas*				*HPT*
	HT																	HD							HTD			JT				
*Substance*	1	2	3	4	5	6	7	8	9	10	11	12	13	14	15	16	17	1	2	3	4	5	6	7	1	2	3	1	2	5	6	
*Halite, NaCl*	X	X		X	X	X	X	X	X	X		X	X	X																	X	
*Thenardite, Na*_*2*_*SO*_*4*_								X					X	X		?	X	?						?								X
*Sodium hydrogen sulfate, Na*_*3*_*H(SO*_*4*_*)*_*2*_														?																		
*Barite, Ba*_*2*_*SO*_*4*_			X																													
*Gypsum, CaSO*_*4*_*·2H*_*2*_*O*																														X		
*Quartz, SiO*_*2*_											X					X		X	X	X	X	X	X	X	X	X	X					
*Calcite, CaCO*_*3*_							X											X	X					X								X
*Vaterite, CaCO*_*3*_																								X								
*Calcium oxalate, CaC*_*2*_*O*											X							X	X		X											
*Whewellite, CaC*_*2*_*O*_*4*_*·H*_*2*_*O*																X		X	X	X	X	X	X	X	X	X	X					
*Weddellite, CaC*_*2*_*O*_*4*_*·2H*_*2*_*O*											X											X			X	X						
*Plagioclase*																			X	X	X		X				?					
*Alkali feldspar*																				X	X						?					
*Muscovite/illite, KAl*_*2*_*(AlSi*_*3*_*O*_*10*_*)(OH)*_*2*_																								X								
*Rutile, TiO*_*2*_			X																													
*Sodium 1-pentanesulfonate, C*_*5*_*H*_*11*_*NaO*_*3*_*S*		?			?			?		?		?			?																	
*Sodium 4-methylpentanoate, C*_*6*_*H*_*11*_*NaO*_*2*_														?																		
*Sodium stearate, C*_*18*_*H*_*35*_*NaO*_*2*_														?																		
*Acetanilide, C*_*8*_*H*_*9*_*NO*											X																					
*ẟ-D-Mannitol, C*_*6*_*H*_*14*_*O*_*6*_																												X	X			
*Methyl-3,4-dinitrobenzoate, C*_*8*_*H*_*6*_*N*_*2*_*O*_*6*_																							X									
*Sucrose, C*_*12*_*H*_*22*_*O*_*11*_														?									X									

Question marks represent tentative identification of the crystalline phases

XRPD revealed that quartz and calcium oxalates (CaC_2_O_4_·xH_2_O) are present in all henna powder samples. Calcium oxalates play an important role in the calcium biomineralization processes that occur in plants [24]. In fact, these mineral phases not only constitute a bioavailable reserve of calcium in plants, but are also responsible for calcium regulation and homeostasis, and have a functional role in its excretion [24]. Angiosperms are known to form the calcium oxalates whewellite and weddelite, but also calcium carbonate polymorphs calcite and vaterite, which may explain the identification of these phases in samples HD-1, HD-2, and HD-7 [25]. Calcite is also used as a disintegrant, diluent, binder, and opacifier in the pharmaceutical industry [26].

**Fig. 3:**
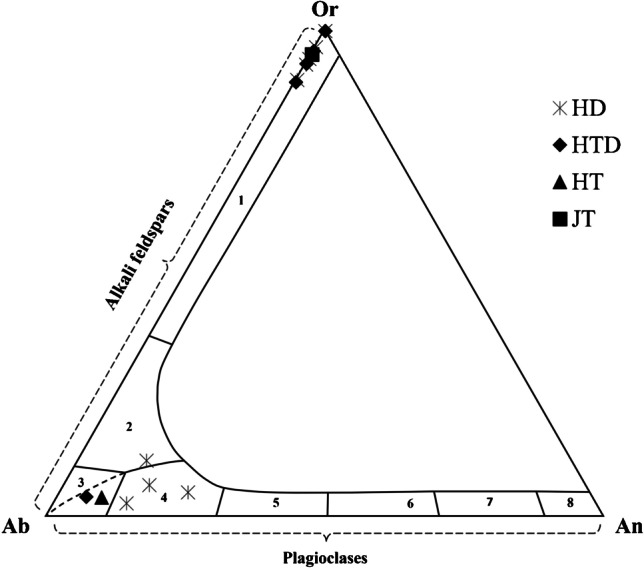
Feldspar diagram. 1: sanidine; 2: anorthoclase; 3: albite; 4: oligoclase; 5: andesine; 6: labradorite; 7: bytownite; 8: anorthite. Plagioclases and alkali feldspars were identified in several henna powder samples (HD and HTD), while albite was identified in henna paste HT-11 and sanidine in jagua JT-2

The identification of quartz in all henna powder samples, alkali feldspars and plagioclases in a large number of these samples, and muscovite/illite in sample HD-7 can be explained by the use of pigment extenders [27, 28] or be related to the manufacturing process of these commercial products. Quartz crystals have previously been identified in different colored henna samples [29], and their presence was justified as a constituent of several natural extracts such as horsetail (E*quisetum hyemale*) which are mixed with the henna powder to care for both hair and skin due to their rich mineral content. Plagioclase, specifically oligoclase, and the alkali feldspars anorthoclase, sanidine, and orthoclase were also identified in henna powder samples HD-1, HD-4, HD-6, HD-7, HTD-1, HTD-2, and HTD-3 by VP-SEM–EDS (Fig. 3). Methyl-3,4-dinitrobenzoate and sucrose were identified by XRPD in sample HD-6. According to the Cosmetic Ingredient Database, CosIng [30], sucrose is used as an ingredient in cosmetic products with moisturizing, skin conditioning, or soothing functions.

As seen in Table 2, quartz and calcium oxalates were only identified by μ-XRD in henna paste samples HT-11 and HT-16. Halite was detected in all samples with the exception of HT-3, HT-11, HT-15, HT-16, and HT-17. Halite is used as an emulsifying, anticaking, and isotonic agent to manufacture pharmaceuticals [26], but also as a viscosity regulator [31] and thickener [32] in the cosmetics industry. Thenardite, a sodium sulfate commonly used as a viscosity-increasing agent in the production of cosmetics [33], was identified in samples HT-8, HT-13, and HT-14. Sodium 1-pentanesulfonate was tentatively identified in samples HT-2, HT-5, HT-8, HT-10, HT-12, and HT-15. Sodium 4-methylpentanoate, sodium stearate, sodium hydrogen sulfate, and sucrose were also tentatively identified in sample HT-14, while acetanilide was detected in sample HT-11. Some of these substances have specific functions as ingredients in cosmetic products [30]. Sodium stearate is employed as a cleanser, surfactant, emulsifier, or viscosity controller; sodium hydrogen sulfate as a buffering agent; and acetanilide is used as a fragrance or perfumer. Rutile and barite were identified only in sample HT-3, which is consistent with the high Ti and Ba contents detected by h-XRF. Barite, the naturally occurring form of BaSO_4_, is a white compound commonly used as a pigment extender; rutile, on the other hand, is a tetragonal titanium oxide frequently referred to, in its synthetic form, as titanium white due to its use as a pigment [34]. Moreover, the identification of rutile and barite in sample HT-3 by μ-XRD is consistent with the white color displayed by this henna paste.

Gypsum was identified in the jagua paste sample JT-5, while ẟ-D-mannitol was identified in paste samples JT-1 and JT-2. However, it was not possible to identify the crystalline phases found in jagua paste sample JT-3 and in jagua powder JT-4 using the PDF‐2 database, which suggests they may be organic compounds. Gypsum is frequently used in the manufacture of cosmetics [35] and as a diluent and binder in the pharmaceutical industry [26]. Mannitol has functions as a binder, fragrance, humectant, moisturizing, or skin conditioning [30] when it is added as an ingredient in cosmetics.

Thenardite and calcite were identified by μ-XRD in the plant-based temporary tattoo sample HPT.

To summarize this section and in view of Table 2, differences and similarities that stand out when comparing the different types of samples considered can be mentioned. Hennas for tattooing (HT) are based on sodium salts, mainly halite and in some cases sodium sulfate, while those for hair dyeing (HD) have a quartz base and a high presence of calcium salts such as oxalates or carbonates. As previously indicated, the real hennas pastes (HT-11 and HT-16) in terms of the presence of active ingredients [17, 18] are similar to the solid hennas and this is also shown in Table 2, with the presence of quartz and calcium oxalates. Furthermore, those hennas for both purposes (HTD) have a similar composition to the dyes (HD), so according to this content, these two types of henna samples would not be differentiated. However, the general composition makes it possible to clearly distinguish the paste samples (HT) from the solid ones (HD and HTD). In addition to other sporadic or specific presences of each sample already mentioned above, in the case of jaguas, their inorganic component is minimal, which also differentiates them from the henna samples. In some jaguas (JT-3 and JT-4), no crystalline phase is found, so it can be affirmed that they are completely organic materials. Finally, the same applies to the sample that does not belong to any of the groups, HPT, which has a low inorganic content and is therefore closer in content to the jaguas. However, it should be noted that the active compounds of jaguas were not found [17, 18], so this initially suggests that it is not a sample based on jagua extracts.

### Characterization by VP-SEM–EDS

**Fig. 4:**
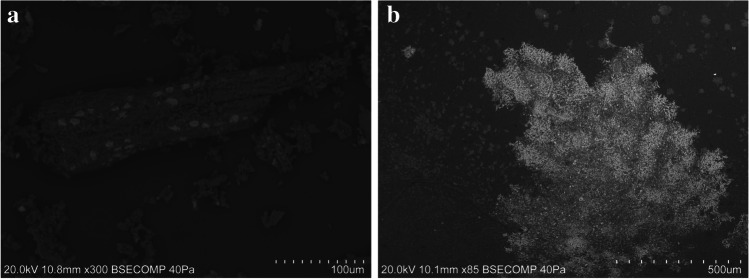
SEM images of the samples analyzed which display different morphological features according to their classification as powder or paste. **a** SEM micrograph of henna powder HD-1 showing great heterogeneity and the presence of plant tissue remains; **b** SEM micrograph of the black henna paste HT-10

VP-SEM–EDS was used to perform a microstructural and microanalytical characterization of the samples. Point analysis and elemental mapping were also used to identify their chemical composition. The analysis revealed the differences between the two groups of samples considered; in general, the powdered samples were more heterogeneous than the pastes and contain visible plant tissue remains (Fig. 4). However, henna paste samples HT-11 and HT-16 were found to be both morphologically and chemically similar to the powdered hennas.

**Fig. 5:**
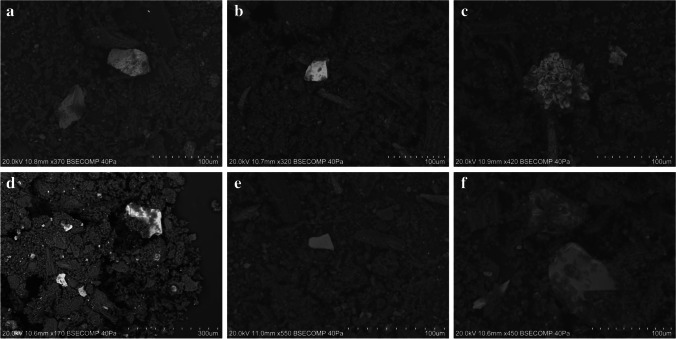
Other mineral phases found in the powdered henna samples analyzed: Fe–Mn oxide (**a**), zircon (**b**), iron sulfide (**c**), various aluminosilicates (**d**), iron-rich grain (**e**), sodium sulfate (**f**)

Powdered henna samples present heterogeneous granular textures, with angular, sub-angular, and sub-rounded grains being mixed with plant tissues and unrecognizable carbon-rich structures (Fig. 4a). The composition of the grains is very diverse; however, silica-rich and calcium-rich grains were ubiquitous, which is consistent with the identification of quartz and calcium oxalates in all powder henna samples by XRPD. As previously mentioned, both feldspars and plagioclases were identified by VP-SEM–EDS in a significant number of samples (Fig. 3) and were often accompanied by other undetermined aluminosilicate phases (Fig. 5). Zircon, calcium phosphate, sodium sulfate, iron sulfide, and iron-rich grains were also found in several powdered hennas (Fig. 5).

**Fig. 6:**
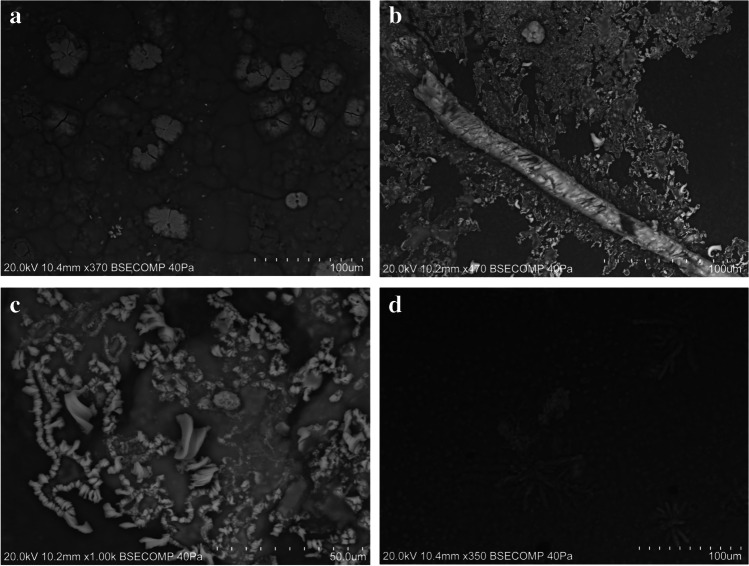
Crystalline aggregates found in the paste hennas: crust-like (**a**), acicular (**b**), rosary (**c**), dendritic (**d**) forms

**Fig. 7:**
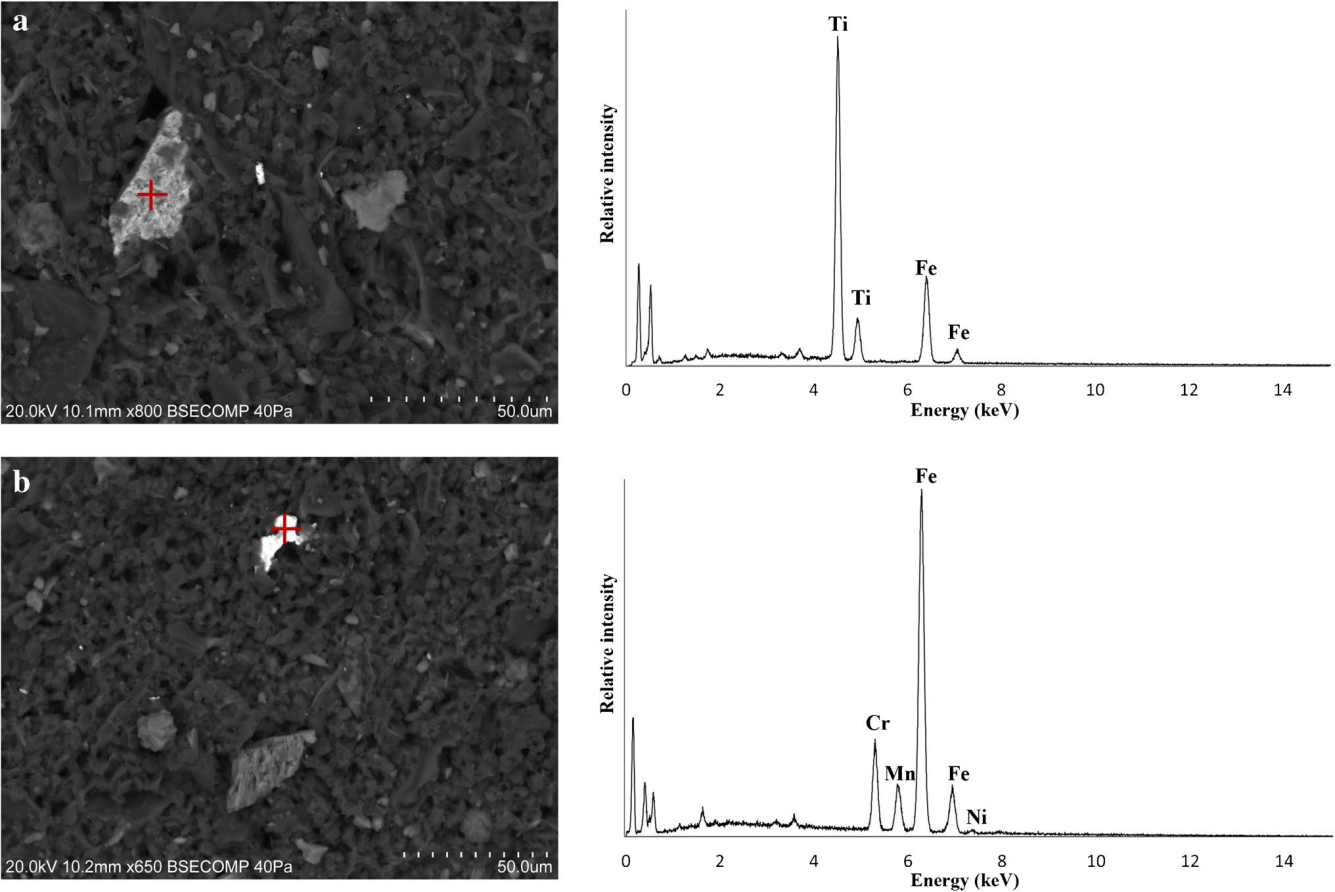
Iron–titanium-rich grains (**a**) and iron-rich grains also containing significant amounts of Cr, Mn, and Ni (**b**) found in henna paste sample HT-11

In general, henna pastes consist of granular, fibrous, or cubic crystals, frequently aggregated to form dendritic, rosary, acicular, or crust-like structures, dispersed in a carbon-based matrix (Figs. 4b and 6). The crystals found are most likely the sodium salts, halite, and thenardite, given their chemical composition, and the identification of these compounds in the henna paste samples by μ-XRD. Samples HT-11 and HT-16 are different from the remaining henna pastes, displaying a more heterogeneous composition with angular and sub-angular grains with variable composition. Silica-rich and calcium-rich grains were found in sample HT-16, which are most likely quartz and calcium oxalate grains, given the identification of these mineral phases by μ-XRD. Albite was identified in sample HT-11 (Fig. 3), along with iron–titanium grains (Fig. 7) and iron-rich grains that also contain significant amounts of Cr, Mn, and Ni (Fig. 7). Transition metals are frequently adsorbed or incorporated into the structure of iron oxide [36] phases that can be difficult to identify by XRD when copper radiation is employed [37], which may explain their absence in the diffractograms acquired. As expected, given the results obtained by h-XRF and μ-XRD, sample HT-3 is composed of a mixture of Ti- and Ba-rich grains.

**Fig. 8:**
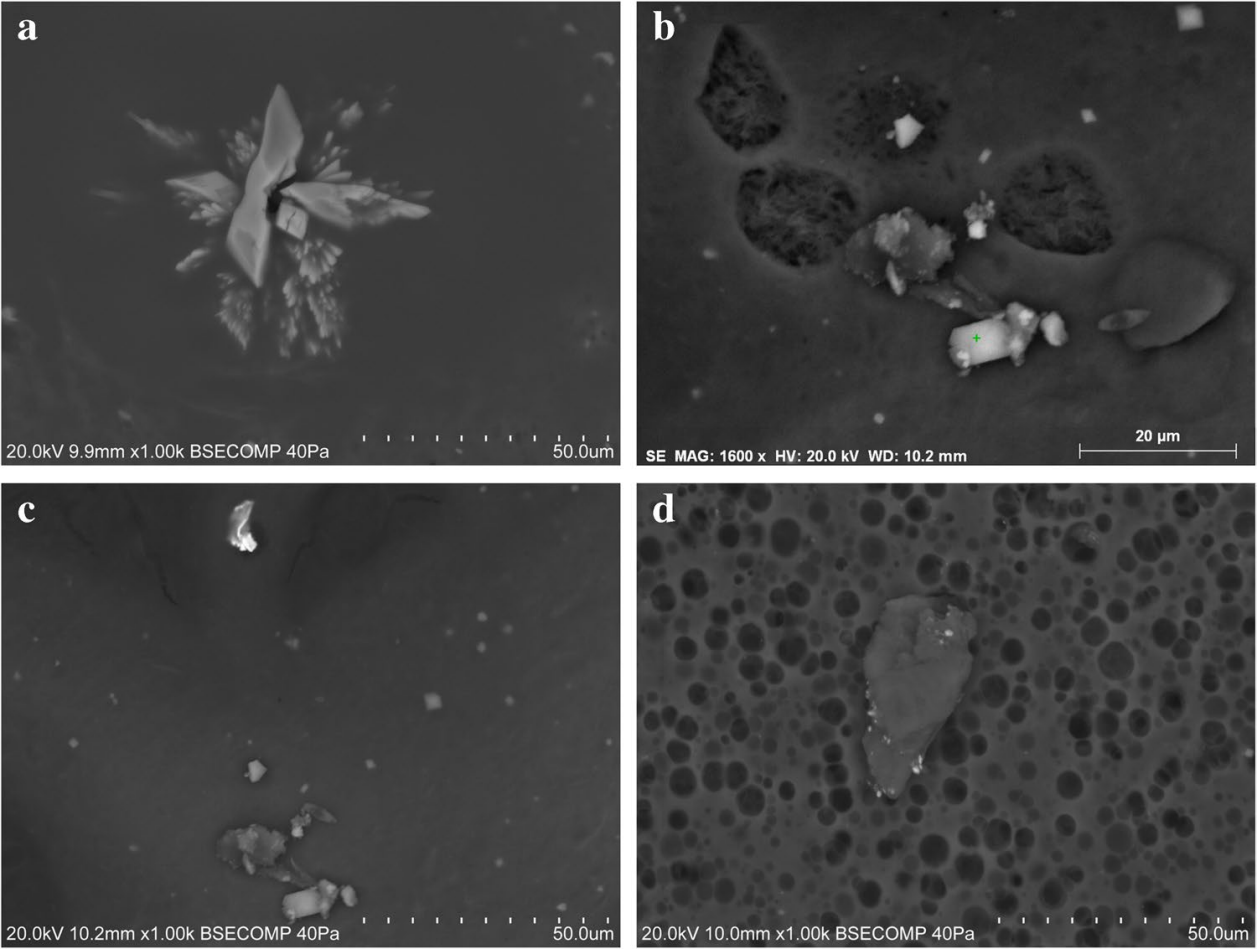
Examples of particles found embedded or on the surface of the jagua samples analyzed: euhedral and prismatic gypsum crystals displayed in a radial arrangement found in sample JT-5 (**a**); prismatic crystals composed of Ca and S (**b**) and grain enriched in Fe, Cr, and Ni (**c**) found in sample JT-6; and zinc-rich particles identified in sample JT-3 (**d**)

Jagua samples JT-1, JT-2, JT-3, JT-5, and JT-6 have carbon-based matrixes with microcrystalline or cryptocrystalline textures. Grains, crystals, or aggregates can be found embedded or on the surface of the C-based matrix. Calcium-rich and silica and aluminum-rich grains can be found in all samples, and sanidine was identified in sample JT-2 (Fig. 3). Euhedral and prismatic crystals displayed in radial arrangement composed of Ca and S were found in sample JT-5 (Fig. 8), which is consistent with the identification of gypsum in this paste by μ-XRD. Prismatic crystals composed of Ca and S were also found in sample JT-6 (Fig. 8), along with grains enriched in Fe, Cr, and Ni (Fig. 8), while zinc-rich particles were uncovered in sample JT-3 (Fig. 8). JT-4, a powdered jagua, is heterogeneous, consisting of angular and sub-angular grains and aggregates enriched in Si, Al, Fe, or Ca.

**Fig. 9:**
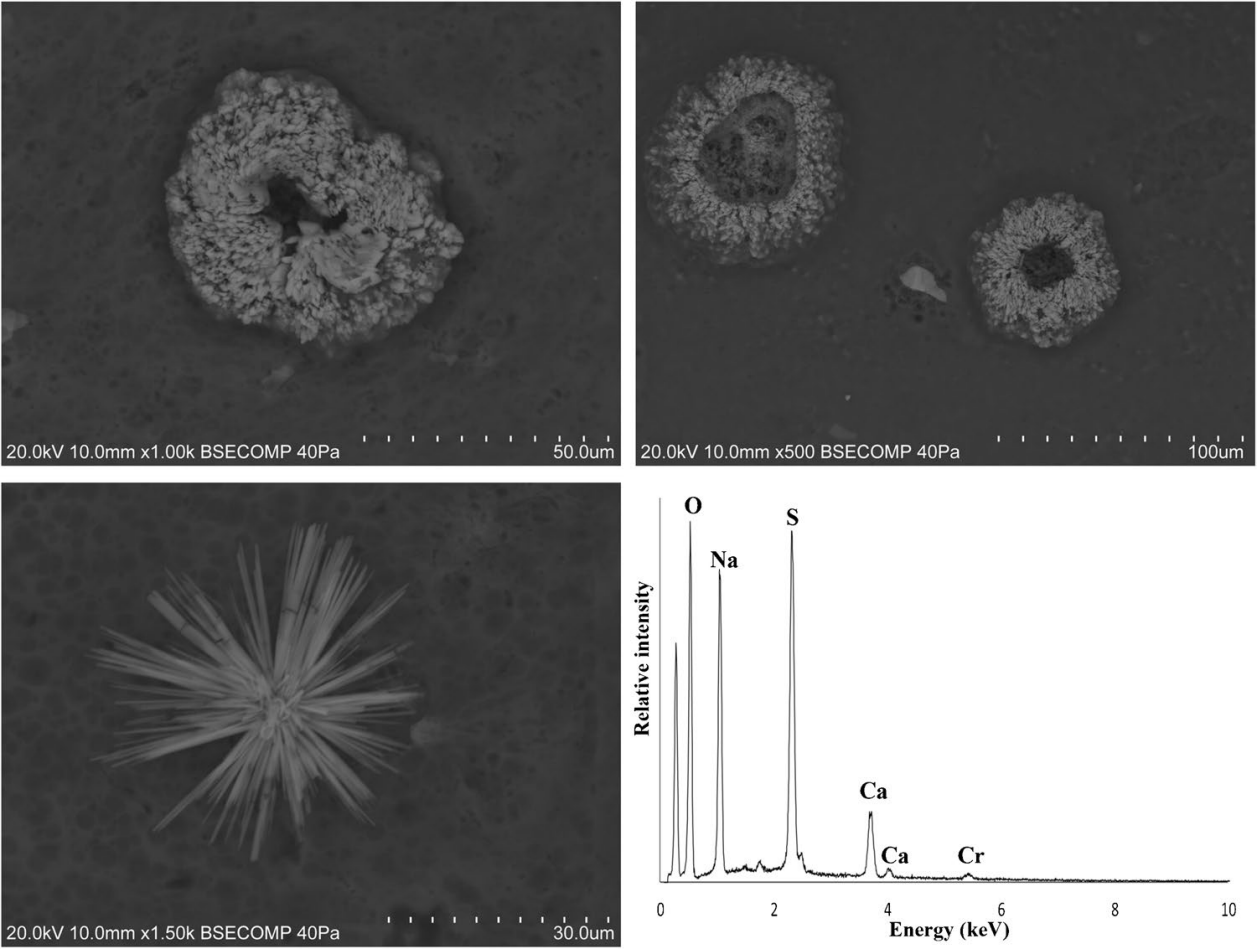
Donut-shaped crystal aggregates of thenardite (top) and a crystalline aggregate formed by acicular grains displayed in an acicular fashion where chromium was detected along with Na, Ca, and S (bottom) found in sample HPT

The plant-based temporary tattoo (sample HPT) has donut-shaped crystal aggregates composed of Na and S embedded in a carbon-rich matrix, which is consistent with the identification of thenardite in this sample by μ-XRD. Chromium was also found in several grains dispersed throughout the matrix, including in a crystalline aggregate formed by acicular grains displayed in a radial fashion (Fig. 9). High Ti values were also found in a number of grains analyzed.

Crystalline aggregates containing elements such as Al, S, K, Ca, Si, and Mg have previously been found in temporary paint-on-tattooing and hair-dyeing henna commercial products [29, 38]. Significant Pb contents have also been previously found in henna products [29], but this toxic element was not detected in any of the samples analyzed in this study.

### Further chemical characterization and European regulation

**Fig. 10:**
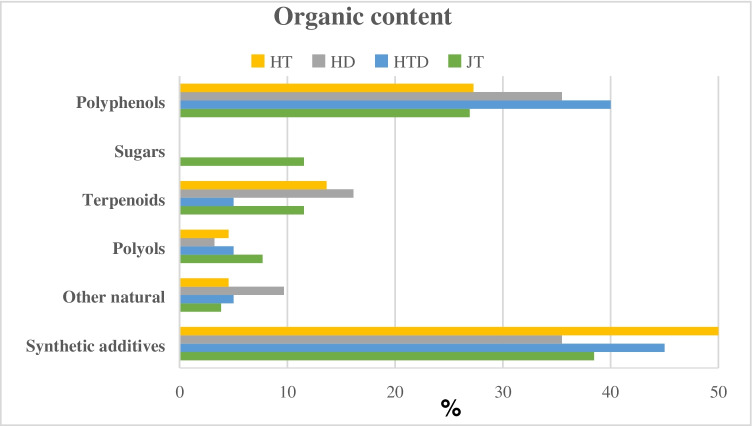
Contribution of organic composition to the different types of samples: JT (jagua for tattooing), HT (henna for tattooing), HD (henna for hair dyeing), and HTD (henna for both purposes)

In previous studies, the authors carried out the analytical characterization of the organic composition of jagua and henna samples [17, 18]. Compounds of natural origin, mainly secondary metabolites associated with the vegetable origin of the raw material (polyphenols, terpenoids, sugars, and polyols), have been quantified. In addition, synthetic compounds such as colorants, plasticizers, pH adjusters, preservatives, and UV filters were also found in the samples. Figure 10 summarizes the organic composition of the samples analyzed. Reviewing the data, the presence of sugars in the jaguas and, in general, the level of synthetic additives in the samples could be noted. Most of these substances have specific functions and consequently are permitted as ingredients in cosmetic products, according to the European regulation [12].

The techniques applied in the present study made it possible to detect the presence of other substances present in the tattoo and dye samples that are also allowed in cosmetics with specific uses such as coloring agents or UV filters. In this context, several compounds have been observed that can be included here as permitted colorants under established purity criteria as aluminum (CI 77000) and copper (CI 77400) as white and brown colorants, respectively. In addition, compounds such as titanium dioxide (CI 77891), barium sulfate (CI 77120), calcium carbonate (CI 77220), or naturally hydrated aluminum silicate containing Ca, Mg, quartz-sand, mica, etc. as impurities (CI 77004), which were detected in several samples analyzed, are allowed to be used as white colorants. In the specific case of Zn, the regulation does not include the use of elemental Zn as an ingredient, but it restricts certain compounds for its application depending on the type of product and lists some others that are permitted, such as zinc oxide (CI 77947) also as a white colorant.

The content of metals in the samples can have two possible origins. Metals are naturally associated to the metabolism of the plant and what it takes from the soil where it is grown. In addition, the presence of metallic impurities can be due to technically unavoidable levels that come mainly from the use of allowed dyes such as CI 75470 (Natural Red 4) in some HT and HTD samples and CI 15510 (Acid Orange 7) in several HT samples [18]. However, certain substances prohibited in cosmetics were also found in henna and jagua products. Chromium or P and its acids, salts, or other derivatives cannot be present in cosmetic products, although they were detected in the samples analyzed by h-XRF or VP-SEM–EDS. Therefore, these products do not comply with the current cosmetic regulations.

The complementarity of this research to the previous ones can be highlighted here since it allows uncovering the presence of prohibited elements in cosmetic products and associating other contents with previously found substances. Thus, in addition to the uncertainty about the composition of jagua and henna tattoos and dyes due to insufficient information, a further concern is that uncontrolled commercial products with unknown quantities of prohibited elements are present on the market and may pose a health risk.

## Conclusions

The combined use of the three complementary techniques h-XRF, XRD, and VP-SEM–EDS allowed the characterization of the natural pigment-based temporary tattoo and dye samples from an inorganic point of view, which completed previous work focused on the organic composition, and contributed to gaining further knowledge regarding the mineralogical and chemical composition of the samples, since the literature on the application of the abovementioned techniques to henna samples is quite limited and non-existent in jagua samples.

After applying the proposed multi-analytical approach, the results obtained indicate that henna and jagua temporary products have a very heterogeneous physical and chemical composition and that many of them contain significant amounts of toxic elements such as Cr or P, whose presence is not permitted according to current European legislation, and suggesting that these products lack any quality control during product sourcing and manufacturing and that they are produced from low quality and even impure substances. Since their prolonged use can pose a health risk because they directly contact the skin or hair and most of them are sold unlabeled without any information, strict regulations must be established to comply with potential contaminant limits and good manufacturing practices in beauty cosmetics. In this sense, the information obtained will allow us to contribute to the regulatory point of view to support the implementation of a specific regulation for jagua and henna temporary tattoos and dyes to protect consumer health, especially when children could be involved. In addition, the fact that they are so readily obtained from Internet sources is particularly worrisome; regular inspection of the market should be carried out to detect any fraudulent or dangerous product.

## Supplementary Information

The online version contains supplementary material available at ….

Supplementary file 1 (PDF 659 KB)
