# Supplementary material for: Understanding the chemical and mineralogical composition of commercial henna and jagua tattoos and dyes—a multi-analytical approach
Source: Anal Bioanal Chem. 2022 Jul 13;414(20):6233–46. doi: 10.1007/s00216-022-04194-1 (PMC9314285; doi:10.1007/s00216-022-04194-1)
Supplement: Supplementary file 1 — (PDF 659 KB) [file 216_2022_4194_MOESM1_ESM.pdf]

Supplementary Information

Understanding the chemical and mineralogical composition of commercial henna and jagua tattoos and dyes–a multi-analytical approach

Laura Rubio[1, 2,*], Mafalda Costa[3,*], Pedro Barrulas[3], Marta Lores[2], Carmen Garcia-Jares[1, 2], Cristina Barrocas-Dias[3, 4]

[1]CRETUS, Department of Analytical Chemistry, Nutrition and Food Science, Universidade de Santiago de Compostela, E-15782, Santiago de Compostela, Spain

[2]Laboratory of Research and Development of Analytical Solutions (LIDSA), Department of Analytical Chemistry, Nutrition and Food Science, Universidade de Santiago de Compostela, E-15782, Santiago de Compostela, Spain

[3]HERCULES Laboratory, University of Évora. Évora, Palácio do Vimioso, Largo Marquês de Marialva 8, 7000-809, Évora, Portugal

[4]Chemistry and Biochemistry Department, School of Sciences and Technology, Rua Romão Ramalho, 59, 7000-671 Évora, Portugal

* Corresponding authors e-mail: laura.rubio.lareu@usc.es, mcosta@uevora.pt

Content:

Table S1 *Pictures of the analyzed products. HT, henna tattoo; HD, henna dye; HTD, henna tattoo-dye; JT, jagua tattoo; HPT, herbaceous plant tattoo*

Sample code	*Brand*	*Picture*
**HT-1 to HT-9**	Golecha (Indian origin)	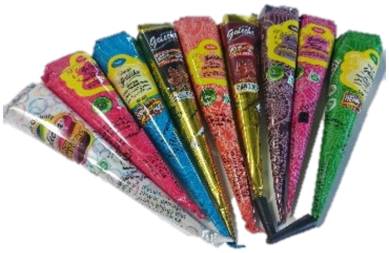
**HT-10 to HT-12**	Golecha (Indian origin)	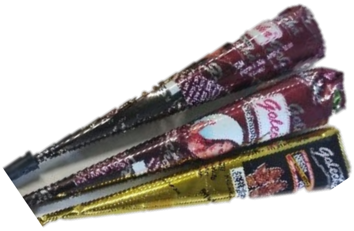
**HT-13 and HT-14**	Shumaila (Pakistani origin)	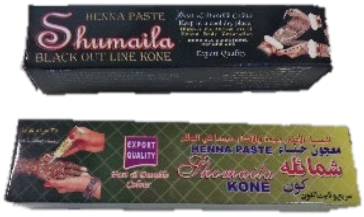
**HT-15**	Kashmir (Indian origin)	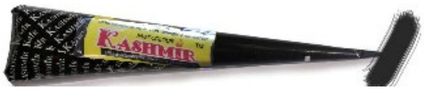
**HT-16**	- (Indian origin)	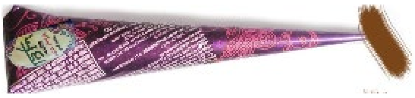
**HT-17**	Golecha (Indian origin)	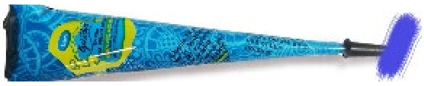
**HD-1**	Radhe Shyam (light brown hair color)	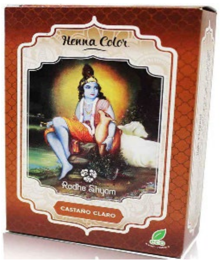
**HD-2**	Radhe Shyam (dark brown hair color)	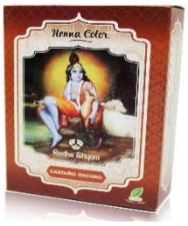

HD-3

Radhe Shyam (black hair color)

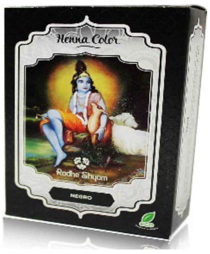

HD-4

Radhe Shyam (auburn hair color)

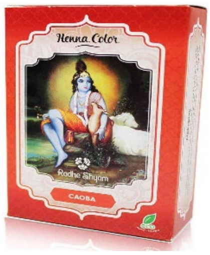

HD-5

Radhe Shyam (natural cooper hair color)

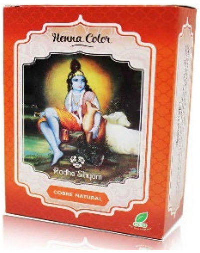

HD-6

Radhe Shyam (deep blonde hair color)

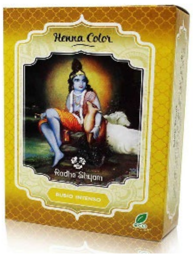

HD-7

Pride of India (Indian origin, red hair color)

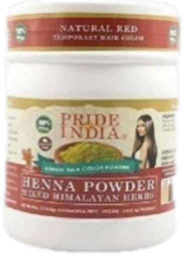

HTD-1

Henna Sahara Tazarine (Marrakech origin)

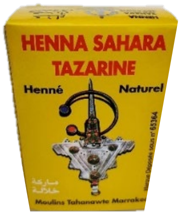

HTD-2

Naturel Anytime

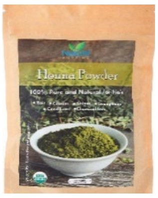

**HTD-3**	Naturel Anytime	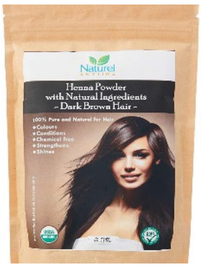
**JT-1**	Easy Ink	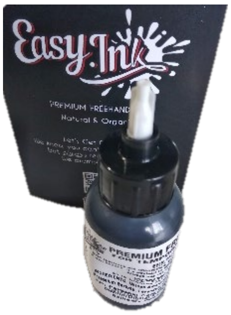
**JT-2**	Jagua Tattoos	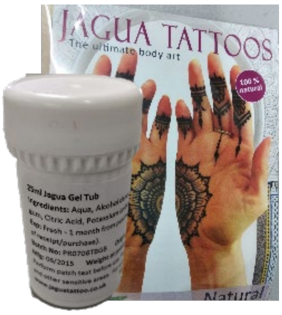
**JT-3**	Mystic Ink	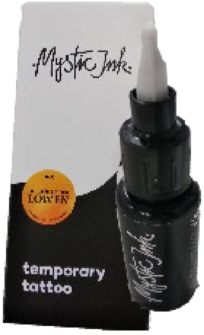
**JT-4**	Jacquard	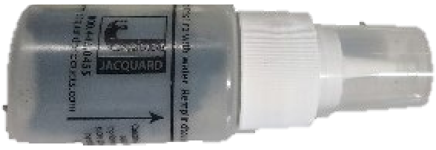
**JT-5**	TruuMe	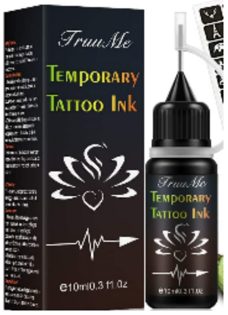

**JT-6**	PDH	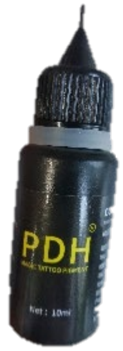
**HPT**	Xing long tattoo	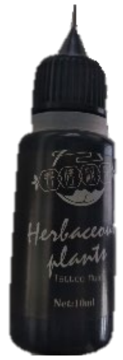
